# Pathogenic *Rickettsia* spp. as emerging models for bacterial biology

**DOI:** 10.1128/jb.00404-23

**Published:** 2024-02-05

**Authors:** Brandon Sit, Rebecca L. Lamason

**Affiliations:** 1 Department of Biology, Massachusetts Institute of Technology, Cambridge, Massachusetts, USA; Geisel School of Medicine at Dartmouth, Hanover, New Hampshire, USA

**Keywords:** *Rickettsia*, obligate intracellular bacteria, bacterial pathogen, model organism

## Abstract

Our understanding of free-living bacterial models like *Escherichia coli* far outpaces that of obligate intracellular bacteria, which cannot be cultured axenically. All obligate intracellular bacteria are host-associated, and many cause serious human diseases. Their constant exposure to the distinct biochemical niche of the host has driven the evolution of numerous specialized bacteriological and genetic adaptations, as well as innovative molecular mechanisms of infection. Here, we review the history and use of pathogenic *Rickettsia* species, which cause an array of vector-borne vascular illnesses, as model systems to probe microbial biology. Although many challenges remain in our studies of these organisms, the rich pathogenic and biological diversity of *Rickettsia* spp. constitutes a unique backdrop to investigate how microbes survive and thrive in host and vector cells. We take a bacterial-focused perspective and highlight emerging insights that relate to new host–pathogen interactions, bacterial physiology, and evolution. The transformation of *Rickettsia* spp. from pathogens to models demonstrates how recalcitrant microbes may be leveraged in the lab to tap unmined bacterial diversity for new discoveries. *Rickettsia* spp. hold great promise as model systems not only to understand other obligate intracellular pathogens but also to discover new biology across and beyond bacteria.

## INTRODUCTION

## THE NEED FOR “NON-MODEL” MODEL BACTERIAL SYSTEMS

Classical features of model bacteria, especially dominant workhorse species like *Escherichia coli,* include their ease of manipulation for rapid experimentation and their ability to proxy the inaccessible and/or complex biology of another species. An oft-referenced quote from Jacques Monod about model bacteria roughly translates to the following: “What is true of *E. coli* is true of the elephant” (1). While Monod was originally referring to the central dogma, we now know that what is true for the prototypical *E. coli* lab strain K-12 is not even necessarily true of all K-12 lineages, much less wild *E. coli* or other physiologically and genetically distinct species (2–4). Similar limitations apply to other common bacterial model species, such as *Bacillus subtilis* and *Caulobacter crescentus* (5, 6).

There is increasing interest in the development of “non-model” organisms that may not conform to the features of traditional models but rather have great potential as research tools due to their biological diversity (7, 8). In the context of bacteria, particularly those that are pathogenic, non-model species can be rich sources of new biology, often stemming from their remarkable capacity to adapt to the restrictive and frequently hostile niches found within eukaryotic hosts. Here, we examine the development of non-model bacteria—pathogenic *Rickettsia* spp.—as emerging model systems. *Rickettsia* is a Gram-negative, alphaproteobacterial genus whose constituent species cause a wide spectrum of globally distributed vector-borne human vascular diseases of considerable public health concern. All *Rickettsia* spp. are considered obligate intracellular microbes, meaning that they cannot be cultured axenically and undergo their lifecycles within host and/or vector eukaryotic cells. This and other challenges discussed below have greatly hindered our understanding of these bacteria. However, over a century of work on *Rickettsia* spp. has revealed numerous aspects of their lifecycle and pathogenesis that position these species as potential models for bacteriology and understanding host–microbe interactions. The tremendous genetic and phenotypic diversity within the *Rickettsia* genus also provides multiple perspectives to understand specialized and novel bacterial functions associated with vector and host adaptation.

## PATHOGENIC *RICKETTSIA* SPP.: PAST AND PRESENT

Historically, pathogenic *Rickettsia* spp. were broadly classed into two groups that caused similar, but clinically distinguishable, febrile vascular illnesses (rickettsioses). The spotted fever group (SFG) causes a spectrum of tick-borne illnesses marked by fever, myalgia, and a characteristic spotted rash and eschar (9). The most severe SFG rickettsiosis is Rocky Mountain spotted fever (RMSF), which can progress from the initial onset of signs of illness like fever and rash to organ failure and death within a matter of days (9). The second major group of pathogenic *Rickettsia* spp. belong to the typhus group (TG) and cause similar febrile illnesses known as epidemic (louse-borne) and endemic/murine (flea-borne) typhus (10). *Rickettsia* is the major genus of what are known colloquially as “rickettsial” species, which comprises related arthropod-borne obligate intracellular bacterial pathogens in the Rickettsiales. This order includes *Orientia tsutsugamushi* (scrub typhus)*, Anaplasma phagocytophilum* (human granulocytic anaplasmosis), and *Ehrlichia chaffeensis* (human ehrlichiosis), which all have unique pathogenic traits despite relatively close genetic relationships (11). Although nomenclature can be variable, here, we use the term “*Rickettsia* spp.” to specifically refer to species within the *Rickettsia* genus and “rickettsia/rickettsiae” to generically refer to bacteria in the Rickettsiales.

**Fig 1:**
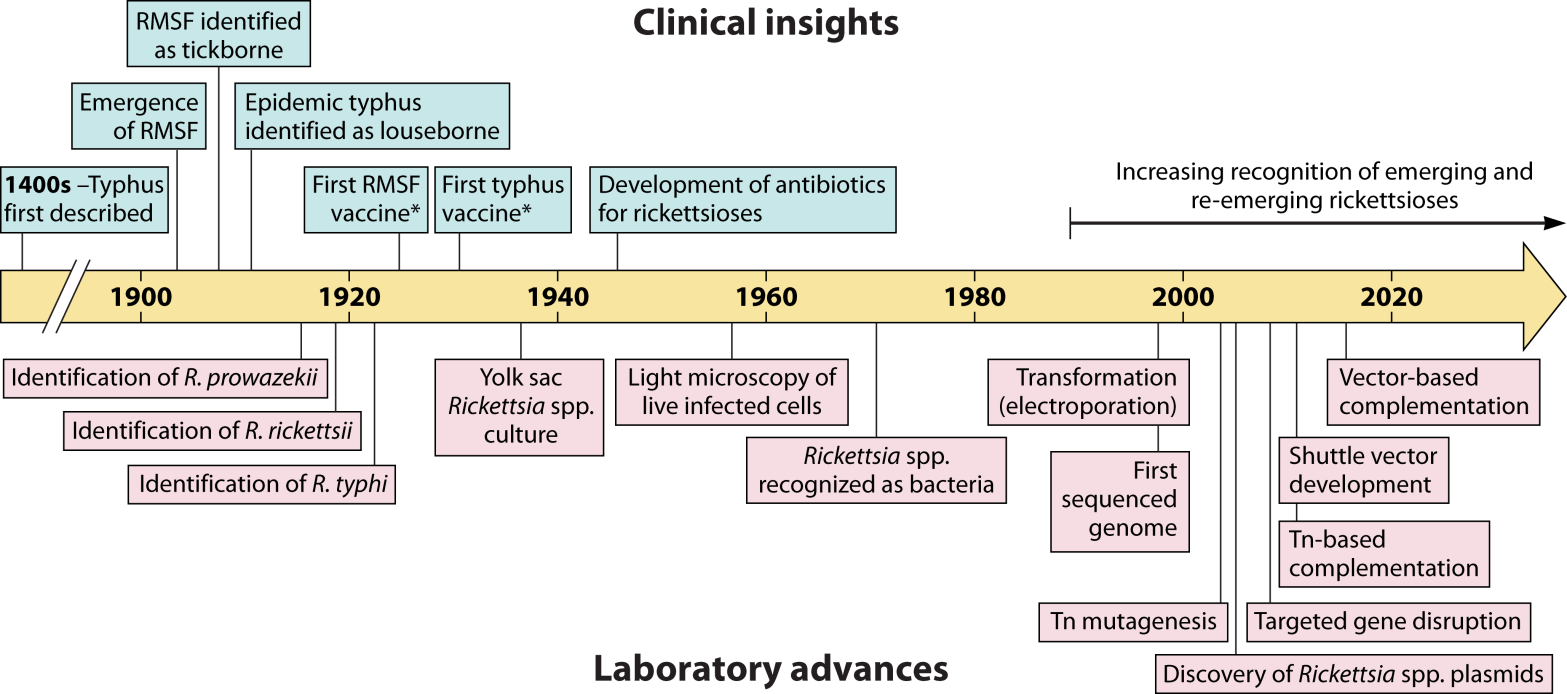
Clinical and experimental milestones in *Rickettsia* research. Tn, transposon. ^*^Although vaccine candidates have long existed for rickettsioses, there are no approved SFG or TG vaccines in use today.

The history of SFG and TG rickettsioses, which dates back to the ~1400s, has been extensively documented (12–15). The first *Rickettsia* spp. to be described was *Rickettsia rickettsii,* the pathogenic agent of RMSF (Fig. 1). Pioneering work by the American bacteriologist Howard T. Ricketts in the early 1900s established that this disease was tick-transmitted, making RMSF the first known human tick-borne bacterial illness (16). Ricketts’ development of a guinea pig RMSF model, which is still in use today, provided an essential experimental tool and led to key insights into immunity (17, 18). Ricketts also described small diplococcoid organisms in infected animal tissues and suggested that RMSF was a microbial disease, although he was unable to culture the agent and fulfill Koch’s second postulate before his death from typhus in 1910 (19). His hypothesis was ultimately fulfilled by Burt Wolbach in 1919, who further characterized the pathogen, demonstrated the vascular nature of RMSF, and proposed the name *Dermacentroxenus rickettsi* (now *R. rickettsii*) in honor of Ricketts (20).

Contemporaneously with the emergence of RMSF in the USA, the etiologic agents of epidemic and endemic typhus were identified as *Rickettsia prowazekii* and *Rickettsia typhi,* respectively (Fig. 1). The French microbiologist Charles Nicolle was the first to report that epidemic typhus, the cause of many notable outbreaks throughout military and societal history, was louse-borne (14, 21). *R. prowazekii* was formally identified in 1916 by the Brazilian researcher Henrique da Rocha Lima, who named the organism after the late Ricketts and Stanislaus von Prowazek (another casualty of typhus) in recognition of their combined contributions to the discovery of this pathogen (22). By 1920, endemic typhus was recognized as a separate, milder clinical entity than epidemic typhus and *R. typhi* (formerly *R mooseri*) was identified (13). Despite similar disease presentations, *R. prowazekii* and *R. typhi* have contrasting epidemiology, with the former causing sustained outbreaks in overcrowded conditions where lice are present and the latter more frequent in urban environments where human contact with murine hosts and fleas is more likely (10).

**Fig 2:**
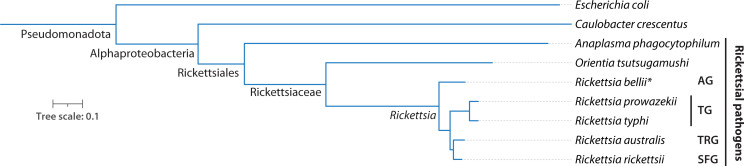
Phylogenetic position of *Rickettsia* spp. within bacteria. The tree displays whole genome phylogenies using the indicated species from GTDB (26). The tree was generated with phyloT (https://phylot.biobyte.de/) and plotted with iTOL (https://itol.embl.de/). Note that TRG, AG, and SFG contain additional species than those depicted. ^*^R. bellii is considered non-pathogenic in humans.

Extensive work following the identification of the major pathogens *R. rickettsii, R. prowazekii,* and *R. typhi* revealed that *Rickettsia* is a highly diverse and phylogenetically complex genus, covering a wide spectrum of bacterial adaptations to confined host and vector intracellular niches (Fig. 2). Although *R. typhi* and *R. prowazekii* remain the only TG members, there are now ~20 described SFG species that cause similar pathologies but have distinct geographic distributions and tick species tropism (23). Some examples are *Rickettsia conorii* (boutonneuse fever), *Rickettsia africae* (African tick-bite fever), and *Rickettsia japonica* (Japanese spotted fever). There are also now two additional recognized groups of *Rickettsia* spp. The transitional group (TRG) includes *Rickettsia akari* (rickettsialpox) and *Rickettsia australis* (Queensland tick typhus), which cluster between SFG and TG species (24) (Fig. 2). The ancestral group (AG), which includes *Rickettsia canadensis* and *Rickettsia bellii*, is considered to be non-pathogenic and represents the most evolutionarily distant *Rickettsia* members (25) (Fig. 2).

Compared to other infectious diseases, global estimates of *Rickettsia* spp. burden are lacking, likely due to ambiguous disease presentation and insufficient surveillance. The development of effective antibiotics, sanitation, and vector control programs sharply curtailed rickettsial disease burdens. Consequently, associated research funding and interest post-World War II also declined (12). However, in the USA, where rickettsioses are nationally notifiable conditions, the incidence of SFG and TG infections has risen dramatically during the 21st century (27–31). There have also been particularly severe RMSF outbreaks in Arizona and northern Mexico (32–36). Although serological studies can be confounded by factors like cross-reactivity, work in diverse locales revealing high seropositivity in rural areas and frequent undiagnosed rickettsioses in patients with febrile illnesses of unknown etiology support the idea that these diseases are on the rise at a global scale (37–41). New human pathogenic SFG *Rickettsia* spp. have been described as recently as 2010 (42), and as much as half of the global population may now be at risk of an SFG rickettsiosis, a trend that has been attributed to climate change-dependent vector range expansion (43, 44). Rickettsioses are additionally considered neglected infectious diseases, where research efforts are not proportional to the corresponding disease burden (45). Collectively, these lines of evidence demonstrate that rickettsioses are both emerging and re-emerging, motivating additional basic studies of *Rickettsia* spp. to power diagnostic and therapeutic developments.

## CHALLENGES TO OVERCOME IN THE STUDY OF *RICKETTSIA* SPP.

The field of rickettsiology arose concurrently with the development of modern bacteriology in the late 19th and early 20th centuries (Fig. 1). So why has our understanding of *Rickettsia* spp. not kept pace with other intracellular bacterial pathogens like *Salmonella enterica* subspp. and *Listeria monocytogenes,* not to mention species like *E. coli*? While safety concerns limit the use of some *Rickettsia* spp. to biosafety level 3 facilities, the extensive characterization of other level 3 pathogens like *Mycobacterium tuberculosis* suggests this factor does not account for the research lag. There is also a widening usage of safer surrogate *Rickettsia* spp., such as the mildly pathogenic SFG species *R. parkeri* (46)*,* to model their more dangerous relatives. Instead, it is the obligate intracellular nature and lack of genetic tractability of *Rickettsia* spp., discussed below, that continue to obstruct their use as model organisms.

### Lack of an axenic culture system

The greatest experimental obstacle to studying *Rickettsia* spp. is their obligate intracellular lifestyle and the lack of an axenic (free-living) culture system. Efforts to cultivate rickettsiae have persisted since RMSF emerged. Ricketts developed the first “isolation” method by serial passaging of *R. rickettsii* between guinea pig hosts (17). In the 1930s, rickettsiae cultivation in the egg yolk sac was reported by Herald Cox, followed later by tissue dissociation and culture methods that enabled animal host-independent rickettsiae growth (12, 47). Early studies of bacteria isolated from infected host cells revealed key factors like glutamate and potassium needed to maintain pathogen metabolism, permitting the development of buffers that preserve limited extracellular viability and enable the use of frozen bacterial stocks (48–50). Two of these buffers, SPG (48) and K-36 (50), are still widely used today. The adoption of yolk sac and tissue culture systems were key inflection points in the field and dramatically accelerated the pace of research on *Rickettsia* spp. biology. Importantly, these systems enabled the resolution of the long-standing taxonomic question of whether these pathogens were bacteria, viruses, or another microbial entity altogether. This involved a body of evidence that revealed *Rickettsia* spp. have traditional bacterial cell envelopes, divide by binary fission, are sensitive to antibiotics, and undergo active metabolism when isolated from the host—advances that enabled the framework of bacteriology to be applied to these species (51, 52). Although the propagation of *Rickettsia* spp. in tissue culture is now routine, there are still major limitations inherent to this growth system. These include the difficulty of obtaining pure, high-yield bacterial preparations free of host cell debris and organelles, variability of culture infectivity and phenotypes, spontaneous loss of virulence during serial passage, and high usage of consumables. Additionally, the relatively long time (days–weeks) needed to produce large quantities of bacteria or isolate clonal strains means that rapid method development and optimization are not as feasible in *Rickettsia* spp. as in axenically culturable species.

### Poor (but improving) genetic tractability

The powerful genetic toolkits enjoyed by most other bacteriologists typically rely on axenic culture-state bacteria and, thus, are not accessible to researchers working on *Rickettsia* spp. Commonly used engineering techniques like allelic exchange or recombineering are hampered in *Rickettsia* spp. by low transformation efficiency and limited selection and counterselection methods that work in the eukaryotic cytosol. Without the ability to readily make targeted genetic changes, experiments on virulence factor biology that are common in other pathogens, for example, determining necessity and sufficiency to fulfill molecular Koch’s postulates (53), have remained relatively rare. Alternative approaches, like antibody-mediated or small molecule inhibition (54), peptide nucleic acid-based knockdown (55), and heterologous expression techniques (56), can be useful in combination, but they are limited by incomplete inhibition, potential off-target effects, and overexpression artefacts.

Decades of work have improved, but not unlocked, genetic tractability in *Rickettsia* spp. (Fig. 1). Electroporation of *R. rickettsii* freshly isolated from host cells was first reported in 1998, a decade later than the same advance in *E. coli* (57, 58). This method remains the only known way to transform *Rickettsia* spp. (59). Selectable antibiotic markers and fluorescence reporters of non-rickettsial origin were then shown to be functional in transformed SFG and TG rickettsiae (60–62). By the mid-2000s, the discovery of naturally occurring plasmids in several *Rickettsia* spp. opened the door to versatile plasmid-based manipulation, and engineered shuttle vectors that can be used in both SFG and TG *Rickettsia* spp. were recently reported (24, 63–70). Applications of replicative plasmids in *Rickettsia* spp. are still nascent—for example, plasmid-based mutant complementation was not demonstrated until 2016 (71), and extrachromosomal expression of a fluorescently tagged endogenous protein was only reported in 2023 (72).

Early studies establishing electroporation also demonstrated that plasmids and linear DNA could recombine into SFG or TG rickettsiae genomes (57, 60, 62). However, only three subsequent studies, all using different approaches, have reported recombination-based gene disruption. In 2009, linear homologous DNA was used to delete a predicted secreted phospholipase in *R. prowazekii* (73). Next, an intron-based technique frequently used to engineer the obligate intracellular pathogen *Chlamydia trachomatis,* Targetron, was reported in 2015 to successfully delete a major outer membrane protein in *R. rickettsii* (74). Most recently, fluorescent reporter-assisted allelic exchange was used to delete a putative regulator of *R. rickettsii* intracellular motility (75). While these efforts were critical for evaluating the roles of specific gene products in infection, no additional strains generated by any of the above methods have been reported. Thus, while recombination-based engineering methods are biologically possible in *Rickettsia* spp.*,* their current applicability appears limited, possibly due to a combination of technical (e.g., low electroporation efficiency) and biological (e.g., defense mechanisms against foreign DNA) factors.

Compared to targeted methods, random mutagenesis approaches are more common and are often used to generate attenuated strains of *Rickettsia* spp. The oldest random mutagenesis method is spontaneous mutation, an unavoidable consequence of the serial passage of an obligate intracellular bacterium. One notable example is the *R. prowazekii* vaccine strain Madrid E, which was attenuated through >250 yolk sac passages and led to the identification of a methyltransferase virulence gene candidate (76–78). A more defined alternative to spontaneous mutagenesis is the use of transposons, which insert at random genomic sites in the host bacterium. Transposons delivered by plasmids or transposomes are viable tools for random mutant generation in both TG and SFG rickettsiae at efficiencies that permit small-scale phenotypic screens (64, 79–86). They are also useful for insertion at presumed neutral genomic sites with a defined payload, such as a complementation allele or tagged protein of interest (87–89). Transposon mutants provide vital loss-of-function strains for experimentation and have enabled both the discovery and functional characterization of loci involved in infection. Currently, however, large-scale pooled transposon screening and next-generation sequencing-based approaches like TnSeq, which are popular in other species (90), cannot be performed in *Rickettsia* spp.

## NOTABLE CONCEPTS IN *RICKETTSIA* SPP. PATHOGENESIS, BIOLOGY, AND EVOLUTION

In spite of the technical constraints associated with *Rickettsia* spp., work from numerous groups has illuminated many aspects of rickettsial biology that comprise a compelling argument for the use of these pathogens as model organisms. Many cell types, including epithelial, endothelial, and immune lineages, can support *Rickettsia* spp. infection in tissue culture. During *in vivo* infection, however, while rickettsiae-containing immune cells can be isolated, vascular endothelial cells are considered the primary pathological target cell type, where sustained infection leads to barrier leakage and impedance of normal vessel function (91). Disruption of vascular integrity accounts for the fever, rash, and most systemic signs of disease observed in patients. The intracellular *Rickettsia* spp. infection cycle is superficially similar to those of other cytosolic pathogens (92). Once *Rickettsia* spp. are delivered to the host by an arthropod vector, cellular infection is initiated by receptor recognition and internalization. Invasion is followed by escape from the initial entry vacuole into the cytosol, the primary replicative niche for the pathogen. Within the host cytoplasm, *Rickettsia* spp. begin to replicate while interfacing with and evading host defenses such as autophagy. Some *Rickettsia* spp. assemble “tails” of the host cytoskeletal protein actin, propelling them around the cell in a behavior termed actin-based motility. At later timepoints, *Rickettsia* spp. exit the primary cell and infect new neighbor cells or transit to distal sites. Detailed reviews of *Rickettsia* spp. virulence, host response, and vector biology have been recently published (11, 93–99). Rather than a comprehensive review, we discuss selected themes that illustrate the multifaceted adaptations developed by *Rickettsia* spp. for their host cytosolic niches.

### Distinct export mechanisms for diverse surface and secreted effector proteins

*Rickettsia* spp. infection broadly relies on surface-exposed and secreted effector proteins with diverse functions, many of which are specifically discussed in later sections. The best-known surface-associated rickettsial virulence factors belong to the surface cell antigen (Sca) protein family, which are restricted to the *Rickettsia* genus and were identified by early phylogenetic studies (100). Some Scas are canonical autotransporters (type 5 secretion systems) that mediate their own trafficking to and insertion in the outer membrane, whereas others lack autotransporter-associated domains and are instead considered to be secreted (71, 101, 102). The accessory domains of each Sca vary considerably, and less than half of the 17 originally identified Sca subtypes have been functionally characterized. Different Scas participate in essentially every infection stage, and some are multifunctional (101). The functions and biochemical features of the Scas have enriched our understanding of how autotransporters and autotransporter-like proteins can promote bacterial pathogenesis.

Other secretion mechanisms, including type 4 (T4SS) and type 1 (T1SS) secretion systems, may mediate *Rickettsia* spp. effector translocation into the host cytoplasm (101). The rickettsial T4SS has been the subject of particular interest, since T4SSs in other intracellular pathogens like *Coxiella burnetii* and *Legionella pneumophila* are critical for effector secretion and pathogen fitness during infection (103). Interestingly, the rickettsial T4SS is not homologous to these deeply characterized T4SSs. Instead, it most closely resembles the *vir* T4SS of the extracellular plant pathogen *Agrobacterium tumefaciens* and is thus known as the *r*ickettsial *v*ir *h*omolog (*rvh*) system (101, 104, 105). *rvh* systems diverge from *vir* T4SSs in several ways. For example, *rvh* systems have a predicted homolog for every *vir* component except VirB5, which is essential for T4SS pilus formation but not substrate transport. Thus, *rvh* systems may secrete substrates not through a pilus contacting a membrane like canonical T4SSs but rather directly into the extracellular space (i.e., the host cytosol). Additionally, unlike the single *vir* coding region in *A. tumefaciens, rvh* loci are scattered throughout the genome and exhibit altered copy numbers (see below) (101). This atypical T4SS organization and structure make *rvh* systems excellent candidates for investigating T4SS modularity and function. Since an *rvh*-null mutant has not been generated in any *Rickettsia* spp. and *rvh* effectors have not been comprehensively identified, the precise role(s) of this secretion system in infection is somewhat of an enigma. T4SSs are found across the Rickettsiales, and further studies of the mechanisms by which *Rickettsia* spp. assemble their host-associated surface proteomes and deliver effectors to the cytosol should reveal new strategies employed by intracellular pathogens to target the host.

### Novel mechanisms for intracellular motility and intercellular spread

Unique molecular features of *Rickettsia* spp. infection are particularly evident in the lifecycle stages involving actin-based motility and cell-to-cell spread. Motility and spread phenotypes and their underlying molecular mechanisms diverge between SFG and TG rickettsiae (106–109). Actin-based motility has been mostly characterized in SFG rickettsiae, which undergo both short-tailed (early) and long-tailed (late) phases of motility and spread directly from cell to cell without host lysis (106, 110). In early motility, SFG rickettsiae use the surface protein RickA to mimic host actin nucleation-promoting factors that stimulate actin polymerization through the activation of the Arp2/3 complex (110, 111). This conceptually resembles the well-characterized *L. monocytogenes* nucleation-promoting factor ActA*,* even though RickA and ActA are not homologous. Later in infection, the Sca family member Sca2 drives the assembly of phenotypically distinct actin tails (110, 112). Sca2 structurally and functionally mimics host formins, which directly nucleate actin filaments, a mechanism not found in any other bacterial pathogen (113–115). In contrast, TG rickettsiae lack RickA and only encode truncated Sca2, potentially explaining why their motility phenotypes differ from SFG species. Understanding why some *Rickettsia* spp. undergo biphasic actin-based motility and the contributions of these mechanisms to infection may reveal new principles of pathogen manipulation of the host cytoskeleton.

In SFG species, after a period of intracytosolic replication and motility, bacteria near the host plasma membrane form protrusions into adjacent host cells (71, 106, 116). Protrusion uptake by host cells and subsequent pathogen escape from the double membrane-enclosed secondary vacuole constitutes a cell-to-cell spreading event. In the model SFG species *R. parkeri,* actin tail loss precedes protrusion formation, in contrast to *L. monocytogenes,* which also undergoes direct cell-to-cell spread but maintains its actin tail in protrusions (71). This raises an apparent paradox—how are pathogen*-*containing protrusions formed and resolved without force from actin-based motility? In SFG *Rickettsia* spp., part of the answer appears to be the Sca family member Sca4, a secreted effector that binds the host junctional protein vinculin and blocks its interaction with α-catenin (71). This relaxes host intercellular tension, facilitating protrusion uptake by adjacent cells. Targeting of vinculin is unique among known mechanisms of pathogen manipulation of host membrane tension (117–119). Rickettsiae-specific innovation is also reflected in pathogen escape from the secondary vacuole, which is mediated by the phospholipase A2-like enzyme Pat1 in *R. parkeri,* unlike the phospholipase C enzymes PlcA and PlcB in *L. monocytogenes* or the type 3 secretion system translocon pore in *Shigella flexneri* (88). The molecular basis of TG rickettsiae cell lysis and spread is less well characterized and may involve a combination of hemolysin and phospholipase activity (120–122). Collectively, the molecular mechanisms of *Rickettsia* spp. actin-based motility and cell-to-cell spread demonstrate a common message: they achieve similar ends as other intracellular pathogens, but they do so through markedly different means.

### Functional and physical interactions with host cell biology

Microbial subversion of host cell biology can reveal as much about the host as it does the bacterium (123). In the cytosol, *Rickettsia* spp. encounter host membrane-bound organelles and cell signaling pathways that are targets for pathogen-secreted effectors. For example, *trans-*Golgi apparatus fragmentation and subsequent disruptions to protein export were recently described in *R. rickettsii* infections (124). This phenotype was attributed to the activity of the ankyrin repeat-containing secreted effector RARP-2, a proposed T4SS substrate that localizes to the endoplasmic reticulum (ER) during infection (125). Another putative T4SS substrate, Risk1, is a phosphatidylinositol-3-kinase (PI3K) that was recently proposed to support both phagosomal escape and autophagy evasion by *R. typhi* (54). A third predicted T4SS substrate is the TG-specific RalF, which alters cellular phosphoinositol metabolism through the host GTPase Arf6 to promote *R. typhi* invasion (126, 127). Interestingly, the phylogenetically distant intracellular bacterial pathogen *Legionella pneumophila* also encodes RalF, but with a different function targeting the host secretory pathway, suggestive of divergent effector evolution (128). Given that intracellular pathogens can encode dozens of secreted effectors and only a handful of *Rickettsia* spp. effectors have been identified, it seems likely that additional secreted effectors and their roles in host cell manipulation await discovery in these bacteria.

*Rickettsia* spp. also display curious localization phenotypes during infection that suggest intimate connections between bacterium and host. Initially observed by Wolbach over a century ago in RMSF patient samples, a common feature of SFG rickettsiae infections is the accumulation of bacteria in the host nucleus (20, 129, 130). Remarkably, intranuclear bacteria can still be associated with actin tails (131, 132). How SFG rickettsiae gain entry to the nuclear compartment and to what end they do so remain open questions. Understanding this process could be informative for understanding other pathogens that also invade the nucleus or alter nuclear function (133). Beyond interactions with the nucleus, *R. parkeri* was recently reported to form putative membrane contact sites with the ER (134). To date, all known pathogen-induced membrane contact sites occur between host-derived membranes. These sites play increasingly recognized roles in infection, including lipid transfer, apposition of secretion systems, and immune response modulation (135). *R. parkeri*–ER contact sites, which were dramatically increased in immotile bacteria lacking Sca2 (134), would represent the first interaction of a host organellar and cytosolic bacterial membrane. Whether such associations are impactful for *R. parkeri* infection remains unclear. Studies of physical associations of rickettsiae with host structures like the nucleus and ER may not only shed light on the pathogen but also uncover aspects of host cell biology responsive to foreign insults.

### Genomic signatures of adaptation to the confined cytosolic niche

*Rickettsia* spp. genomes range from 1.1 to 1.5 Mbp and have undergone substantial re-arrangements, streamlining, and gene degradation (136). In this setting, the retention of any given locus suggests that the function of the encoded gene product supports intracellular fitness, as genes that do not contribute to infection presumably would have been or are being eliminated. This idea is even more salient when genes have increased in copy number. For example, there is frequent atypical T4SS component gene expansion in *Rickettsia* spp., particularly in RvhB6, a secretion channel-associated factor that occurs in up to five copies in some species (105, 137, 138). The presence of five RvhB6 copies in a streamlined genome strongly suggests that these gene products are needed for optimal fitness during infection. Consistent with this idea, an *R. parkeri* strain with an insertion in the fifth paralog, *rvh6e*, was recently reported to have an infection defect in tissue culture (82).

In contrast to gene family expansion, genome streamlining can promote the emergence of proteins that have multiple distinct biological functions, otherwise known as moonlighting. Moonlighting permits genome minimization without loss of functional diversity and has been recognized across bacteria in both infective and non-infective contexts (139, 140). One well-known example of moonlighting in *Rickettsia* spp. is the Sca family member Sca5 (rOmpB). rOmpB has intact autotransporter domains and was one of the first-recognized rickettsial proteins. The first proposed role for rOmpB was as an invasion factor, by binding a surface-exposed fraction of the host DNA repair protein Ku70 (56). rOmpB was then implicated in pathogen serum resistance and evasion of host autophagy (84, 141). The latter function was recently attributed to lysine methylation of rOmpB, which blocks bacterial surface ubiquitination and recognition by host autophagy adaptors (85, 142). This type of autophagy evasion is unique among microbial pathogens, reinforcing the idea that *Rickettsia* spp. are an excellent source of novel host–pathogen molecular interactions.

### Cell biological and metabolic adaptations to the obligate intracellular lifestyle

*Rickettsia* spp. cell envelope biology and metabolism are presumably highly adapted to their obligate intracellular niche. Intriguingly*, Rickettsia* is essentially the only genus in the Rickettsiales that retains canonical bacterial cell wall (peptidoglycan, PG) and lipopolysaccharide (LPS) moieties, both of which are highly immunostimulatory (143). Determining why *Rickettsia* spp. synthesize canonical PG when other Rickettsiales members either encode atypical “intermediate” PG (e.g., *O. tsutsugamushi*) or lack PG entirely (e.g., *A. phagocytophilum*) may reveal different selective pressures these pathogens face inside the host (144). Similarly, LPS is a major *Rickettsia* spp. antigen and appears to have a largely canonical structure, albeit with some variation in lipid A composition and acyl chain length, but knowledge of its precise impact on intracellular infection is lacking (145–152). Recent high-resolution analysis of *Rickettsia* spp. PG (72, 153) and the development of tools for tracking rickettsial growth in host cells (72) are critical advances for addressing these questions.

Many unknowns also remain relating to the metabolic capabilities of *Rickettsia* spp. and which pathogen metabolites are *de novo*-synthesized versus host-derived. *Rickettsia* spp. lack entire metabolic pathways (e.g., glycolysis) that are critical in free-living bacteria (154). Recent metabolic modeling has complemented early empirical studies in generating a global picture of *Rickettsia* spp. metabolic capabilities, but many predictions from these efforts remain to be experimentally validated (49, 51, 155, 156). Because genome minimization creates auxotrophies for host-derived nutrients, targeting non-essential host metabolism to starve the pathogen has been shown to be a potential therapeutic approach (157). The regulation of metabolism by sRNAs in *R. conorii* has recently been reported, suggesting that rickettsiae possess specific regulatory mechanisms to control metabolic activity (158, 159). Progress in this area may inform both translational studies and the development of a complete medium for the axenic culture of *Rickettsia* spp.

### Insights into bacterial evolution and eukaryogenesis from rickettsial genomes

Phylogenetic investigations of *Rickettsia* spp. have empowered wide-ranging discoveries into bacterial evolutionary biology. Early implementation of genotyping (e.g., rRNA loci) alongside phenotyping schemata led to major insights such as the recognition of *Orientia* (previously included in the *Rickettsia* genus) and the identification of the AG rickettsiae (25, 160, 161). Comparative genomics efforts in *Rickettsia felis* in the mid-2000s not only identified the TRG rickettsiae but also uncovered the first conjugative plasmid to be identified in an obligate intracellular bacterium (24, 63). Further work revealed a widespread integrative and conjugative element called the Rickettsiales-amplified genetic element (RAGE) in both *Rickettsia* spp. and *O. tsutsugamushi* (162–164). The presence of conjugative plasmids and RAGEs across the family suggests that lateral gene transfer occurs in the Rickettsiaceae (165), but this has not been experimentally demonstrated. Such investigations would be helpful in determining if conjugation is a viable method for nucleic acid delivery and genetic engineering in these pathogens. Only a handful of obligate intracellular bacteria are known to harbor conjugative elements, suggesting that a deeper study of RAGEs and *Rickettsia* spp. plasmids could reveal how these sequences shift and persist in intracellular species during reductive genome evolution (166).

Another benefit of studying *Rickettsia* spp. genomes has been insights into alphaproteobacterial phylogeny and mitochondrial evolution. Mitochondria are thought to descend from endosymbiotic microbial ancestors, but their phylogenetic position has remained unclear (167). Since the early–mid-1990s, the dominant hypothesis has been that the mitochondrial ancestor closely resembled or belonged to the Rickettsiales (167). This was strongly supported by whole-genome sequencing of *R. prowazekii*, which revealed strong signals of purifying selection and similarities to mitochondria gene content and sequence (168). However, new evidence has re-prioritized an alternative model, where mitochondria instead constitute a sister clade to the Alphaproteobacteria (169–172). In this model, mitochondria and Rickettsiales underwent convergent evolution, rather than diverging from a common intracellular ancestor. Moreover, the discovery of non-intracellular Rickettsiales members from environmental metagenomes suggests that the Rickettsiales ancestor was not itself an obligate intracellular entity and that host association evolved later (173). Although compelling, this new model of convergent mitochondrial evolution has been controversial (174–176), and more studies with additional genomic data are needed to resolve this question. These recent insights show how *Rickettsia* spp. can teach us about the evolution of pathogenic and symbiotic obligate intracellular lifestyles.

## FUTURE DIRECTIONS AND PERSPECTIVES

**Fig 3:**
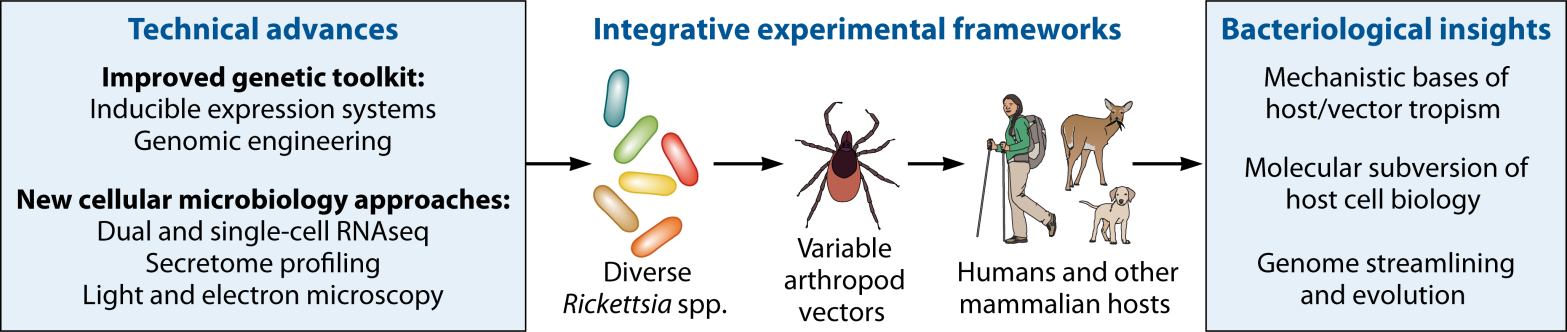
A roadmap for bacteriological research on *Rickettsia* spp. Continued technical development and application of new emerging cellular microbiology tools (left) in experiments designed to capture the diversity of *Rickettsia* spp., their hosts, and their vectors (center) will drastically advance our knowledge of bacterial niche adaptation and fitness mechanisms (right).

Over a century of work on rickettsiae has revealed insights that touch on virtually all aspects of bacteriology. Although these numerous lines of evidence support the idea of *Rickettsia* spp. as model bacteria, it is clear that more work is needed to solidify this idea. We consider here technical development and experimental trajectories that should benefit rickettsiologists and enhance the scope and robustness of new studies on this genus (Fig. 3).

### Strengthening existing experimental approaches in *Rickettsia* spp.

A critical need in the field is an expanded genetic toolkit to investigate drivers of pathogenesis in *Rickettsia* spp. (Fig. 3). For example, further engineering of native plasmids may aid the development of inducible expression system(s), which have not been developed in rickettsiae. Improvement of transformation efficiency coupled with unbiased cataloging of large numbers of transposon mutants shared across laboratories should offer a critical repository for loss-of-function mutants. Because transposon-based techniques cannot target genes essential for obligate intracellular bacterial viability or infection, new tools like inducible or site-directed transposition and CRISPR-mediated transcriptional activation/inhibition are attractive options for application to *Rickettsia* spp. The optimization of selection methods [e.g., flow sorting of fluorescent transformants (177)] is also warranted. Finally, there may be a need to focus efforts from different research groups on a select few *Rickettsia* spp. and strains to accelerate the development of genetic tools, which may then be broadly applied to the diverse members of the genus. Taken together, these steps will buttress and advance the technical approaches used to study rickettsiae in the laboratory, widening the types of questions that can be asked and answered in these species.

The absence of a host cell-free axenic culture system for *Rickettsia* spp. is a major bottleneck to developing efficient genetic manipulation systems for this genus. Although the creation of such a medium is by no means trivial, lessons may be learnt from the related bacterial pathogen *Coxiella burnetii* (Q fever)*,* which was previously considered to be obligately intracellular but was successfully propagated in an extracellular defined media system in 2009 (178). This advance led to a rapid expansion of studies on *C. burnetii* physiology and virulence and the development of robust targeted, random, and plasmid-based genetic modification systems [recently reviewed in (179)]. Thus, axenic culturing of any *Rickettsia* spp. would be a major turning point for the field. It is worth noting, however, that key findings on *Rickettsia* spp. biology from studies in axenic media or non-endothelial tissue culture systems will need to be validated in more physiologically relevant settings (e.g., endothelial cell lines or suitable animal models) to maximize their translational potential.

### Toward high-resolution and high-throughput *Rickettsia* spp. research

Heightened interest in *Rickettsia* spp. as research organisms is now coinciding with the onset of high-throughput approaches, notably dual-RNAseq and -proteomic pipelines, to understand host–microbe interactions (180–183). Although studies in other intracellular pathogens should pave the way for similar approaches in rickettsiae, transcriptomic and proteomic efforts during infection with *Rickettsia* spp. have mainly focused on the host. Studies profiling different *Rickettsia* spp. proteomes (78, 102, 184–186) or using RNAseq to understand pathogen gene regulation (187–190) have been published, but broader comparative and standardized applications of these techniques are warranted. A major limitation is the inefficient enrichment of pathogen biomaterial from host cells. Future work leveraging the ease of working with biosafety level 2 model species like *R. parkeri* and improved selective isolation protocols from infected host cells should facilitate more sensitive detection of transcriptional and proteomic changes during infection (183). Similarly, the adaptation of effector profiling workflows from other pathogens should help reveal the spectrum of secreted *Rickettsia* spp. effector proteins. For example, a cell-selective proteomic strategy originally developed in *Yersinia enterocolitica* was recently deployed in *R. parkeri*, enabling the discovery of multiple new validated secreted effectors (191). Nearly all of these new effectors are specific to the *Rickettsia* genus, underscoring the idea that these pathogens encode unique and highly specialized effector arsenals.

Cell-to-cell variability (heterogeneity) in pathogen gene expression and growth phenotypes between bacteria in the same infected host or host cell can impact infection in diverse contexts, including the establishment and maintenance of infection, defense against immune responses, therapeutic efficacy, and transmission (192–194). Given that phenotypic heterogeneity is now considered common across bacterial pathogens, including intracellular species such as *Salmonella typhimurium* (195) and *M. tuberculosis* (196), *Rickettsia* spp. likely exhibit some degree of heterogeneity during infection. However, the mechanisms underlying such behaviors and their consequences have received little attention in *Rickettsia* spp., as most studies have focused on population-level assays rather than single-cell analysis of host and/or pathogen. Efforts in this regard may reveal new facets of rickettsial physiology as well as therapeutic opportunities. For example, examination of variability in antigen expression during infection could advance both our understanding of *Rickettsia* spp. gene regulation and target prioritization for vaccines that induce optimal protective immune responses against these pathogens. Single-cell transcriptomics (scRNAseq) is rapidly becoming the method of choice to examine heterogeneity, but these questions could also be investigated in *Rickettsia* spp. with fluorescent reporter strains and labeling studies, which have a lower technical barrier (197). Next-generation imaging techniques, such as high-content or super-resolution fluorescence microscopy, may also be useful in understanding heterogeneity in features like *Rickettsia* spp. subcellular localization, cell division, and effector production. These techniques are part of the growing cellular microbiology toolkit, and all represent new ways to visualize rickettsial infection (Fig. 3) (198).

### Harnessing diversity across pathogen*,* vector, and host

The remarkable diversity of *Rickettsia* spp. (Fig. 2) might be considered an obstacle to translating findings in one species across the whole genus. However, studies that incorporate comparisons between rationally selected *Rickettsia* spp. and strains actually have great potential in revealing new biology within the genus. This approach is neatly exemplified by ongoing genomic and phenotypic characterization of two *R. rickettsii* clinical isolates of variable pathogenicity (Sheila Smith and Iowa), which has led to discoveries regarding several effectors mentioned here, including rOmpA, rOmpB, and RARP-2 (75, 124, 199–202). Between-group comparisons have also revealed interesting differences. For example, comparisons between SFG/TG and AG rickettsiae showed differential subcellular localization of secreted effectors like RalF (plasma membrane-localized versus perinuclear) (126) and distinct actin-based motility characteristics potentially mediated by divergent Sca2 variants (203). Broader comparative studies that continue to reach across strain, species, and group divisions will be valuable. The pathogenic SFG and TG groups receive by far the most attention, but the biology of the more evolutionarily distant AG and intermediate TRG species is also crucial to understand, as comparators and in and of themselves.

A related concept is the integration of studies across experimental systems that include both vector and host. Most work currently focuses on either the arthropod vector or the mammalian host—indeed, we focused almost solely on the latter in this review. The behavior of rickettsiae in vectors, where they are usually symbionts and not pathogens, can diverge drastically from that in vertebrate host cells (83, 204, 205). Furthermore, *Rickettsia* spp. can have widely variable vectors despite similar pathogenic mechanisms, including ticks, mites, fleas, and lice. These divergent niches have likely driven different vector adaptation mechanisms in closely related species. Factors critical for *Rickettsia* spp. fitness in one system may not contribute to the other and, thus, may have been overlooked. Recent technical advances in working with arthropod cells, including scRNAseq and CRISPR/Cas genome editing, should ease the integration of vector and host-targeted investigations (206–209). Ultimately, studies that involve both vector and host, or at least test the contributions of specific genes in both systems, will more accurately reflect the biology of these pathogens in the field. This, in turn, may yield progress toward long-standing epidemiological and translational questions such as the molecular bases of vector and host tropism (Fig. 3).

### *Rickettsia*: a model bacterial genus for what?

The trajectory of research on *Rickettsia* spp. provides a fascinating case study of the transformation of pathogens to models (Fig. 1). However, given the challenges of working with these bacteria, can we truly consider them model species? And if so, what precisely do they model? The current lack of genetic tractability and culturability of *Rickettsia* spp. likely precludes their classification as traditional Gram-negative model bacteria like *E. coli*. Rather, *Rickettsia* spp. could be considered models in several different ways. In the narrowest sense, some *Rickettsia* spp. model other *Rickettsia* spp. For example, *R. parkeri* model their more dangerous phylogenetic relatives *R. rickettsii,* presenting a more accessible and tractable experimental system. *Rickettsia* spp. also model the biology of other “unculturable” obligate intracellular bacterial pathogens that share similar niches. Our ability to manipulate *Rickettsia* spp. actually compares favorably with other obligate intracellular pathogens, many of which are even more understudied. For example, at the time of writing, there are no reports of any engineered *O. tsutsugamushi* genomic mutants, even though it is the sister genus of *Rickettsia* (Fig. 2). Finally, and perhaps most broadly, *Rickettsia* spp. may be thought of as a non-traditional model that is particularly well suited for understanding the biology and evolution of bacterial adaptation to stringent and even extreme environments (Fig. 3). The remarkable diversity across the genus (e.g., divergent vectors, hosts, and infection mechanisms) extends this idea, as *Rickettsia* is not a monolithic clade that only models one intracellular lifestyle.

Critically, our review did not cover non-pathogenic species or other areas where *Rickettsia* spp. may also be considered current or potential models, including translational topics (e.g., host innate and adaptive immune responses and development of animal models of infection) and the biology and ecology of these bacteria in arthropod vectors. As these areas of research all suffer similar technical limitations, the continued improvement of methods to manipulate this genus will be instrumental to cementing *Rickettsia* as a model genus for bacterial adaptation and beyond. To revive a 50-year-old lamentation of the lack of young rickettsiologists, “the investigators who face these challenges will find the route interesting and the solutions significant when broadly applied throughout the field of infectious diseases” (210). The wide-ranging insights gained from studying *Rickettsia* spp. emphasize the need for investigators to work with challenging “non-model” bacterial species and capitalize on their diversity to drive discovery in the microbial sciences.
